# Microarray Analysis of microRNA Expression during Axolotl Limb Regeneration

**DOI:** 10.1371/journal.pone.0041804

**Published:** 2012-09-13

**Authors:** Edna C. Holman, Leah J. Campbell, John Hines, Craig M. Crews

**Affiliations:** 1 Department of Molecular, Cellular and Developmental Biology, Yale University, New Haven, Connecticut, United States of America; 2 Department of Chemistry, Yale University, New Haven, Connecticut, United States of America; 3 Department of Pharmacology, Yale University, New Haven, Connecticut, United States of America; Brigham & Women's Hospital - Harvard Medical School, United States of America

## Abstract

Among vertebrates, salamanders stand out for their remarkable capacity to quickly regrow a myriad of tissues and organs after injury or amputation. The limb regeneration process in axolotls (*Ambystoma mexicanum*) has been well studied for decades at the cell-tissue level. While several developmental genes are known to be reactivated during this epimorphic process, less is known about the role of microRNAs in urodele amphibian limb regeneration. Given the compelling evidence that many microRNAs tightly regulate cell fate and morphogenetic processes through development and adulthood by modulating the expression (or re-expression) of developmental genes, we investigated the possibility that microRNA levels change during limb regeneration. Using two different microarray platforms to compare the axolotl microRNA expression between mid-bud limb regenerating blastemas and non-regenerating stump tissues, we found that *miR-21* was overexpressed in mid-bud blastemas compared to stump tissue. Mature *A. mexicanum (“Amex”) miR-21* was detected in axolotl RNA by Northern blot and differential expression of *Amex-miR-21* in blastema versus stump was confirmed by quantitative RT-PCR. We identified the *Amex Jagged1* as a putative target gene for *miR-21* during salamander limb regeneration. We cloned the full length 3′UTR of *Amex-Jag1*, and our *in vitro* assays demonstrated that its single *miR-21* target recognition site is functional and essential for the response of the *Jagged1* gene to *miR-21* levels. Our findings pave the road for advanced *in vivo* functional assays aimed to clarify how microRNAs such as *miR-21*, often linked to pathogenic cell growth, might be modulating the redeployment of developmental genes such as *Jagged1* during regenerative processes.

## Introduction

Although most metazoans are able to repair injured tissues at least to some extent; the capacity to regenerate whole body parts after injury or amputation is limited to a handful of organisms [1]. Uniquely among vertebrates, salamanders (newts and axolotls) can regrow a wide variety of lost or damaged body parts including limbs [2], jaws [3], tail [4], parts of the eye [5]–[7], inner ear hair cells [8], intestines [9] and even large pieces of the heart [10]. In each case, they achieve this by initially dedifferentiating cells in the remaining tissue [11]. One of the most complex types of regeneration is seen in limb regeneration, in which the wound rapidly closes via migration of neighboring epithelial cells and underlying mesenchymal cells change their morphology and proliferate to form a cell mass known as the blastema [12], [13]. Blastemal cells retain a memory of their pre-injury cell and positional identity, which ultimately determines their cell fate and localization in the regrown limb [14].

Over the last century, salamander limb regeneration has been well studied at a gross anatomical level. Although basic similarities to limb development suggest that many genes expressed in developing limbs will be re-expressed during regeneration, little is known about the molecular basis of the regenerative process [15]. Thus, genes whose actions have been demonstrated to be required (although not necessarily sufficient) for the success of the limb regenerative response following blastema induction comprise mostly classic players in limb/appendage development. These include several members of the FGF protein family (i.e., FGF*-1*, *-2*, *-8*, *-10* and *-20*) [2], [16], members of the Wnt signaling pathway [17], [18], *Shh* [19], [20], TGF-beta [21], and several transcription factors such as *Hox* genes including *HoxA* [22], *HoxD* [23], *Msx-1* [24] and *dlx3* [25], among others.

Recognized as important fine regulators of gene expression, microRNAs (miRNAs) are small endogenous noncoding RNA molecules (∼19–25 nt) that bind complementary sequences in the 3′ untranslated region (UTR) of target mRNAs, thus downregulating them at the posttranscriptional or translational levels [26], [27]. Among the diverse roles assigned to miRNAs are the regulation of cellular differentiation [28], proliferation [29] and apoptosis [30]. Not surprisingly, their deregulation has been documented in several diseases including cancer [31], [32]. miRNAs are present across the eukaryotic phylogeny and their striking sequence conservation among taxa has promoted the use of interspecific high-throughput platforms such as miRNA-microarrays. Consequently, it is now feasible to detect and study the involvement of miRNAs in a variety of biological processes and across model organisms, even those without a sequenced genome, such as the axolotl.

Attempts to clone salamander microRNAs have been limited [33], [34] due, in part, to the challenge of assembling generated small sequence reads using the limited and fragmented publicly available newt and axolotl genomic sequences. Nevertheless, alignment comparisons between the set of cloned salamander miRNAs against vertebrate mature miRNAs indicates that in general they share between 90% to 100% sequence identities [33], [34]. This high sequence conservation has allowed the use of interspecific miRNA-microarrays to identify miRNAs involved in some aspects of salamander regeneration such as inner ear hair cell regeneration [35] and lens regeneration in newts [35], [36], as well as axolotl tail regeneration [34].

Herein, we have also taken advantage of the impressive miRNA sequence conservation among taxa and used two different interspecific miRNA-microarray platforms (LC Sciences and Exiqon Life Sciences) to identify differentially expressed miRNAs during limb regeneration in the axolotl (*Ambystoma mexicanum*). Microarray analyses revealed a set of miRNAs that consistently displayed high statistical support and large fold-changes of differential expression between mid-bud regenerating blastemas and mature non-regenerating stump tissue. From this list, *miR-21* was validated by Northern blot and real-time RT-PCR and emerged as a strong candidate for further analyses due to its high and consistent upregulation in regenerating blastemas. We also show by *in vitro* luciferase assays that *A. mexicanum* (“*Amex*”) *Jag1* is directly targeted by *miR-21*. Our findings provide important new insights into the molecular basis of salamander limb regeneration, and implicate *miR-21* in particular as an important component of the genetic machinery in charge of regulating cell lineage determination and proliferation during the limb regeneration process.

## Results and Discussion

In order to identify differentially expressed microRNAs between medium bud regeneration blastemas and non-regenerating limb stumps, we undertook a systematic profiling using two different commercially available microarray platforms: LC Sciences and Exiqon Life Sciences. Both companies received aliquots of the same RNA samples extracted from axolotl tissues (three biological replicates), to be processed according to their own protocols for microarray hybridization, scanning and analysis.

According to LC Sciences, 72 microRNAs were significantly (*p*≤0.01) differentially expressed between 17 days-post-amputation (dpa) regenerating limb blastemas and non-regenerating stump tissue collected from paired samples. According to Exiqon, 47 probes (many of them representing homologous miRs from multiple animal species) were significantly (*p*≤0.001) differentially expressed between the same assessed samples. Multiarray normalized data obtained from both companies can be found as File S1. All microarray data are compliant with Minimum Information About a Microarray Experiment (**MIAME**). The data discussed in this publication have been deposited in NCBI's Gene Expression Omnibus [37] and are accessible through GEO Series accession number GSE29727 (http://www.ncbi.nlm.nih.gov/geo/query/acc.cgi?acc=GSE29727).

**Figure 1:**
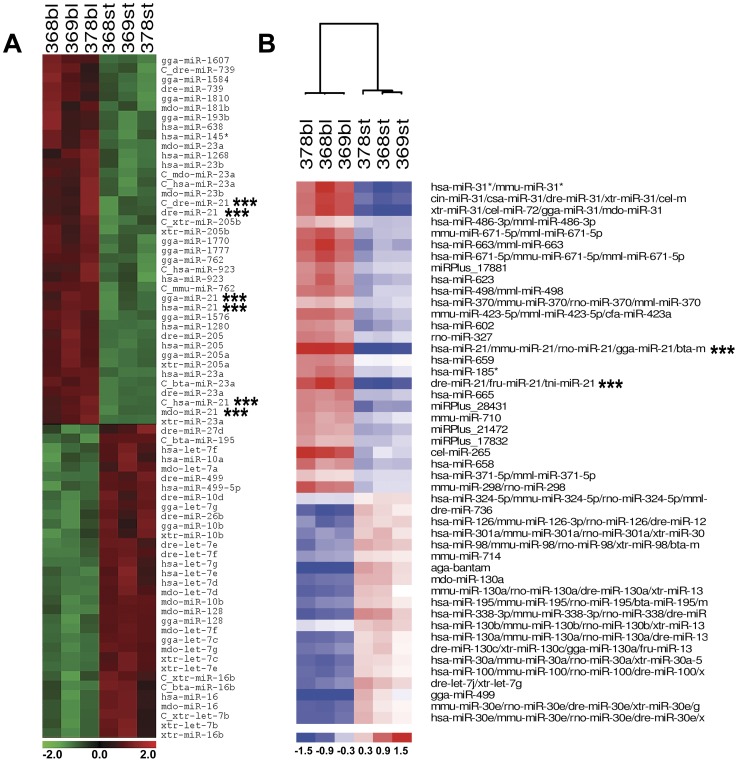
miRNA-microarray profiling of salamander limb regeneration. Clustering analysis performed on log2 (Cy3/Cy5) ratios, which passed the filtering criteria (*p*≤0.01 on **A** and *p*≤0.001 on **B**) using a two-tailed *t*-test between the two groups in the analysis (blastema vs. stump). Heat Map and supervised Hierarchical Clustering of results obtained using LC Sciences (**A**) and Exiqon's (**B**) platforms. Each row represents a miRNA and each column represents a sample. Sample clustering shows that the samples (blastema vs. stump) separate into the two discrete groups. The color scale shown at the bottom illustrates the relative expression level of a miRNA across all samples: red color represents an expression level above mean, green and blue color represents expression lower than the mean. Three consecutive asterisks indicate *miR-21* probes.

Visually, clustering analyses of the microarray results from each company look very similar (**Figure 1**). However, among the most reliable differentially expressed miRNAs (in terms of statistics and fold changes, *p*≤0.01 for LC Sciences, and *p*≤0.001 for Exiqon), only four microRNAs were found to be in common between these two different microarray platforms. Three of these microRNAs (*miR-195*, *miR-499* and *let-7g*) were consistently found to be downregulated in regenerating blastemas. That is, these three miRNAs were found overexpressed in stump tissue as compared with 17 dpa blastemas. Conversely, *miR-21* had an opposite behavior during this regeneration stage and was overexpressed in 17 dpa blastema as compared to stump tissue. It has been suggested that genes (or miRNAs) downregulated during regeneration are likely involved in preserving the terminally differentiated tissue state and will be normally tightly regulated to get a healthy balance between cell growth and abnormal cell proliferation [38]. Accordingly, the upregulation of *let-7g* in mature, non-regenerating axolotl stump tissue correlates with its reported high expression in somatic differentiated cell types [39]. Experimental evidence suggests that *let-7* may, in fact, constitute an anti-stemness factor [40]. In agreement with this, down-regulation of *let-7* members have been previously reported in lens and inner ear hair cell regeneration in newts [35], which implicate them as putative regulators of cell differentiation and its reversal, a crucial phase in salamander regeneration.

The low inter-platform comparability between the microarray results obtained from LC Sciences and Exiqon is not surprising and similar results previously have been reported after systematically comparing microRNA-microarray results from five different companies/platforms [41]. Sources of known variation that could have contributed to the reduced inter-array comparability in our experiments are worth mentioning: the platform-specific probe design based on different releases of miRBase (version 12 for LC Sciences; version 9.2 for Exiqon), the unique labeling methods, different hybridization techniques, particular assumptions made to perform normalization procedures, and differential stringency of detection call.

We performed this microarray profiling of regenerating blastemas at 17 dpa because this is the period when all the animals in our study displayed a medium-bud blastema. At this stage, the blastema resembles a developing limb bud with a large amount of seemingly undifferentiated cells, the nerve dependency of the regenerate is ceasing and little (if any) patterning of new limb structures has begun [42]. Thus, medium bud limb blastema is an attractive stage to identify miRNAs that could be playing important roles in the progression of the regenerate towards proper patterning of the new limb. As noted in **Figure 1** **and** **2**, several probes complementary to mammal, bird and fish *miR-21* were found by both microarray platforms to be the most consistently over-expressed microRNA in mid-bud blastema. According to LC Sciences, when compared to stump tissue, *miR-21* is on average 19 fold over-expressed in mid-bud blastemas (*p*≤0.01); and on average *miR-21* is 8 fold over-expressed (*p*≤0.0001) under the same conditions according to Exiqon's data. Thus, *miR-21* is a strong candidate to be playing an important functional role at this stage of limb regeneration.

**Figure 2:**
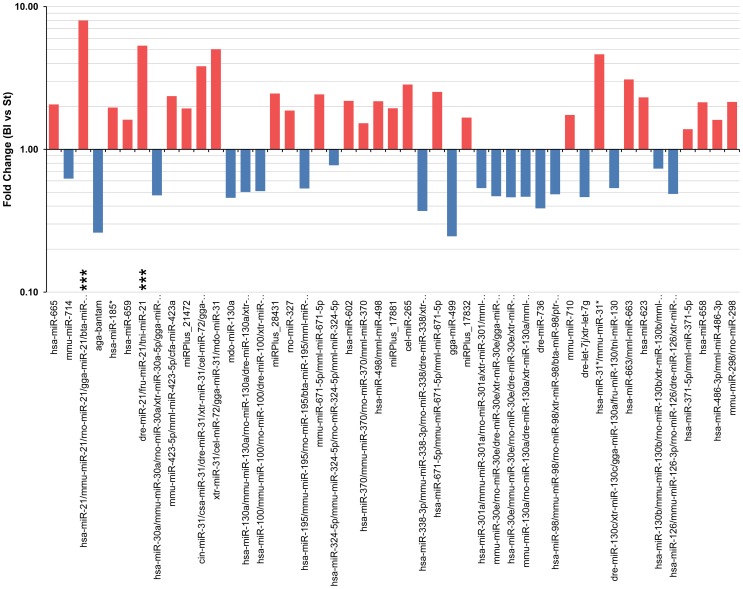
Fold changes of the most significant differentially-expressed miRNAs in salamander limb regeneration. Differentially expressed microRNAs that passed the filtering criteria on variation across samples (*p*≤0.001). Each bar indicates the fold change between blastema (Bl) and stump (St) samples. The y-axis is a log scale. A fold change >1 indicates up-regulation in Bl samples (red bars) and a fold change <1 indicates down-regulation in Bl samples (blue bars) compared to St samples. Three consecutive asterisks indicate miR-21 probes.

Recently a few microRNAs and other small RNAs were isolated, cloned and sequenced from the eyes of adult newts, and *miR-21* was among them [33]. The cloned mature *miR-21* from newt has 100% sequence identity to the mature human *miR-21* [33]. When compared with time-zero samples, the newt *miR-21* was found to be upregulated 1.35-fold at one week of inner ear hair cell regeneration and downregulated 2-fold at 12 days after the initial insult [35]. Interestingly, both of these time-points still represent the time window when cell reprogramming towards transdifferentiation is taking place in this system [35]. However, the changing expression of *miR-21* in opposite directions during this time period might indicate that unrecognized molecular switches have been additionally activated during the reprogramming phase of newt inner ear hair cell regeneration.

To our knowledge, no axolotl *miR-21* (*Amex-miR-21*) sequence is publicly available. However, the reported sequence identity between the newt and human mature *miR-21* indicates that the axolotl mature *miR-21* sequence also may be identical to the human *miR-21 (H. sapiens*, or *hsa-miR-21)*. In fact, *Amex-miR-21* has been found by deep sequencing to be identical to mammal *miR-21* (Karen Echeverri, personal communication). Consequently, the reported upregulation of *miR-21* during inner ear hair cell regeneration [35] and our observation of its over-expression during the mid-bud blastema stage of limb regeneration suggest that *miR-21* is playing an unidentified, yet potentially pivotal, role in salamander regeneration processes characterized by high cell proliferation, low cell differentiation and cell reprogramming phases.

**Figure 3:**
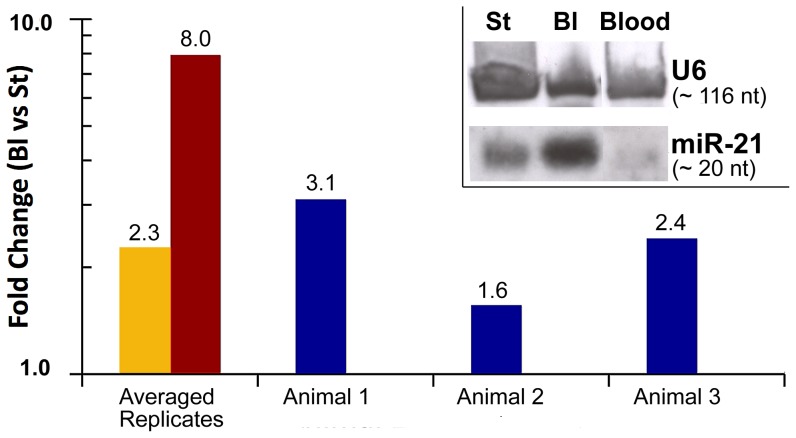
miR-21 expression in axolotl tissues. Quantitative PCR validates *miR-21* microarray results. Two first columns compare the averaged fold change between 17 dpa blastema (Bl) and stump (St) samples for LNA based qPCR assays (yellow bar), and for previous microarray data (red bar). Also, the individual fold changes between Bl and St for the three animals (biological replicates) are shown (blue bars). The relative *miR-21* expression was calculated based on the efficiency corrected ΔΔCt method and normalized with *miR-20a* and *miR-200b*. The y-axis is a log scale. A fold change >1 indicates upregulation in Bl compared to St samples [*p*≤0.03 (qPCR), *p*≤0.0001 (array); two-tailed *t-*test]. **Inset**, illustrates how non-isotopic Northern blot using digoxigenin-labeled LNAs against *hsa-miR21* and *hsa-U6* (control) are useful to detect the axolotl versions of these small RNAs. The axolotl *miR-21* (*Amex-miR-21*) is detected as a band of ∼20 nt which is over expressed in blastema when compared with stump and blood samples.

Given the high sequence conservation for microRNAs across species, the suspected sequence identity between the human and axolotl mature *miR-21* and the particularly good hybridization signal obtained when the axolotl RNA hybridized to the complementary human *miR-21* microarray probes, we attempted to use the *hsa-miR-21* digoxigenin (DIG) -labeled miRCURY Locked Nucleic Acid (LNA) detection probe (Exiqon; Woburn, MA) to perform non-isotopic Northern blot analysis of *miR-21* expression in axolotl blastema, stump and blood. When immobilized axolotl total RNA was hybridized with probes against *hsa-miR-21* and *hsa-U6* (control), discrete bands of approximately 20 nt and 116 nt (respectively) were detected in stump, blastema and blood axolotl tissues (inset on **Figure 3**). Although perfectly equal loading of samples could not be achieved, our result suggests that the axolotl *miR-21* (*Amex-miR-21*) is overexpressed in 17 dpa blastema tissues when compared to non-regenerating stump and blood tissues.

To further validate quantitatively the axolotl microarray and Northern blot data for *miR-21* expression, we used the miRCURY LNA real-time PCR microRNA System (Exiqon). Five putative microRNA endogenous controls were selected from the previous microarray microRNA data. After analyzing their stability using the SLqPCR R-package [43], two of them (*miR-20a* and *miR-200b*) were chosen as endogenous controls for normalization of the efficiency corrected expression data for *miR-21*. Quantitative PCR assays (**Figure 3**) with three biological replicates and four technical replicates validated the previous microarray data and once again demonstrated that *miR-21* is significantly upregulated in 17 dpa blastema tissues when compared with stump tissues.

Among the many predicted targets for *miR-21* reported in the Targetscan 5.1 database [44], [45], *Jagged 1* (*JAG1*) is attractive because of its previously assigned roles in embryonic [46] and limb development [47]. Also, the single recognition site for *miR-21* present in the 3′-UTR of *Jagged1* was previously shown to be targeted by *miR-21* during monocyte-derived dendritic cell differentiation [48]. *JAG1* is a Notch ligand and an evolutionarily conserved target of the WNT/beta-catenin signaling pathway [49]. Given the embryonic lethality of mice homozygous for *Jagged1* loss of function and the essential role of *Jagged1* in remodeling of the embryonic vasculature, an analysis of limb patterning defects in *Jagged1* knockout mice is not possible [46]. While compelling data indicates that *Jagged1* does not participate in early limb pre-patterning events, it is believed to be involved in anterior–posterior axis formation. During limb development, *Jagged1* expression requires a synergistic effect of signals (e.g., Fgfs) emanating from the apical ectodermal ridge (AER) and it is induced by *Shh* signaling via *Gli3* derepression [47]. In 37-day human embryos, *Jagged1* expression has been detected in the distal mesenchyme of limb buds, and mutations in the human *Jagged1* cause the autosomal dominant disorder Alagille syndrome, which affects mainly the liver, heart, vertebrae, eyes and face [50]. Human *JAG1* is expressed in embryonic stem cells, neural tissues, lung carcinoid, gastric cancer, pancreatic cancer, colon cancer, and also in squamous cell carcinoma of the skin, oral cavity, esophagus, head and neck. *JAG1* expression in progenitor cells due to canonical WNT signaling activation induces self-renewal of stem cells via Notch signaling activation. *JAG1*, functioning as WNT-dependent Notch signaling activator, is the key molecule maintaining the homeostasis of stem and progenitor cells [49].

**Figure 4:**
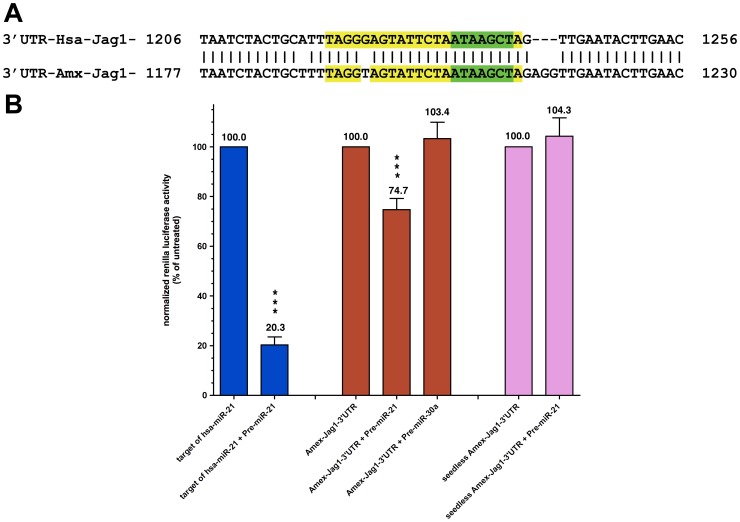
Jagged1 as a putative target of Amex-miR-21. A. Comparison between the human and axolotl miR-21 target sites in their Jagged1 genes. Nucleotide alignment of the *miR-21* target site-containing region present in the 3′-UTR of the human *Jagged1* (*Hsa-Jag1*, NM_000214) and the axolotl *Jagged1* (*Amx-Jag1*, JF907581). The 22 bases comprising the target site for *miR-21* are in yellow except for the 7 bases complementary to the *miR-21* seed region (green color). Vertical bars (|) denote nucleotide identities between the two sequences. The numbers at both sides of the alignment are the nucleotide position on each 3′-UTR. B. *In vitro* luciferase assays testing the effect of *miR-21* on the 3′-UTR of *Amex-Jag1 in axolotl cells*. Results are expressed as percent of co-reporter-normalized *Renilla* activity against reference vectors. Bars denote standard error of mean of the amount of independent assays. Student *t-*test was done and the obtained *p-*values determined that the *Target of Hsa-miR-21* is a good positive control as biosensor for the activity of this microRNA (*p*≤0.005; two-tailed *t-*test). These results suggest that *Amex-Jag1* may in fact be a target for *miR-21* because significant differences were found in the *Renilla* signal recorded from cells electroporated with only the vector containing the 3′-UTR of *Amex-Jag1*, versus the cells that were also electroporated with Pre-miR-21 *** (p≤0.005) but not Pre-miR30a. When the latter experiment was repeated with the mutant, *seedless*-*Amex-Jag1-*3′-UTR, any sensitivity to exogenous *miR-21* was lost.

To determine if the *A. mexicanum Jagged1* (*Amex-Jag1*) gene contains a possible miR-21 target sequence, we cloned the full length 3′-UTR (1,464 bp) of the *Amex-Jag1* gene and submitted its sequence to the dbEST database [accession number: JF907581]. The 3′ UTR of the human and axolotl *Jagged1* are very similar in their full length (75% nucleotide identity). Also, as seen in **Figure 4.A**, the single *miR-21* recognition site present in the 3′-UTR of *Amex-Jag1* is almost identical (only one nucleotide difference) to the single *miR-21* recognition site present in the 3′-UTR of human *Jag1* [accession number: NM_000214], with both having perfect complementarity to the seed region of *miR-21*.

In order to test if *Amex-Jag1* is a target of *miR-21* during axolotl limb regeneration, the full length of the 3′-UTR of *Amex-Jag1* was inserted into the psiCHECK-2 vector (Promega, Madison, WI), which is a dual Firefly/*Renilla* luciferase reporter vector. Other vectors used in the luciferase assays included a biosensor containing just the human target site for *miR-21* (22 nt) and a seedless mutant of the Amex-Jag1-3′UTR vector lacking the 7 nt seed recognition site (green nucleotides in **Figure 4.A**).

All of our assays were performed on AL-1 cells, which are an axolotl dermal fibroblast line [21]. It has been shown that *miR-21* levels can be increased *in vitro* by transfecting miRNA mimics known commercially as Pre-miRs [51], [52]. Pre-miRs are small, chemically modified double-stranded RNA molecules designed to mimic endogenous mature miRNA molecules [53]. As seen in **Figure 4.B**, electroporation of AL-1 cells with Pre-miR-21 suppresses by nearly 80%. the activity of the *Renilla* luciferase reporter containing the known target of *hsa-miR-21* (blue bars), thereby validating the assay system. Interestingly, electroporation with Pre-miR-21 also suppresses the levels of *Renilla* luciferase that incorporates *Amex-Jag1* 3′UTR into the transcript (red bars), albeit to a lesser extent – about 25% inhibition (*p*≤0.005; two-tailed *t* test). A similar statistically significant decrease in luciferase activity (∼20%) was recently reported when the *hsa-Jag1* 3′UTR was proven to be targeted by *hsa-miR-21* [48]. This miRNA-mediated suppression via the Amex-Jag1 3′UTR is specific for miR-21, as electroporation with the structurally unrelated Pre-miR-30a fails to inhibit reporter activity. Furthermore, sensitivity to miR-21 is lost in the variant Amex-Jag1 3′UTR reporter lacking the miR-21 seed sequence (pink bars). Taken together, the reporter data clearly demonstrate that the miR-21 target sequence found in the 3′UTR of Amex-Jag1 is specifically recognized by miR-21 and that such an interaction results in a reduction in gene expression, as it does in humans.

**Figure 5:**
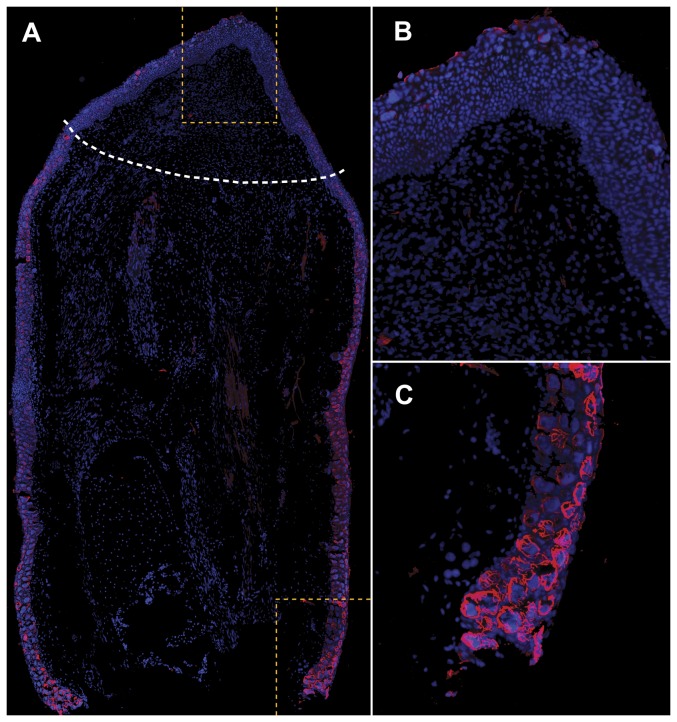
Amex-Jag1 expression in regenerating axolotl limb. Longitudinal tissue section of a 17 dpa regenerating limb immunostained with human Jagged1 antibody. **A.** Higher Jagged1 immunoreactivity (red) is detected in the stump tissue proximal to the animal body (down) than in the mid-bud blastema area (up). White dashed line indicates amputation plane. **B.** A magnification of the blastema area enclosed in yellow dashes shows how Jag1 immunoreactivity is scarce in mid-bud blastema. **C.** Strong Jag1 immunoreactivity is detected in the membrane of epidermal cells in non-regenerating stump. Cell nuclei have been stained with DAPI (blue) present in the mounting media.

If *Amex-Jag1* is in fact being targeted by *miR-21* during limb regeneration, we would expect to see reduced expression of the Jag1 protein in mid-bud blastemas where *miR-21* is upregulated, and higher Jag1 expression in the stump region. As shown in **Figure 5**, Jag1 is expressed especially in epidermal cells both in mature and regenerating epidermis. However, Jag1-expressing cells are significantly more abundant proximal to the animal body. Although **Figure 5** might suggest that Jag-1 is expressed significantly more on the right side compared to the left side in regenerating limbs, in fact, we have seen regenerating limbs with slightly higher Jag-1 expression on the right side, on the left side, and evenly distributed between both sides. Independent of which side of the stump shows higher Jag-1 expression, this Jag-1 immunoreactivity is always significantly higher in stump when compared to blastema tissue as shown in **Figure 5**. Weaker immunoreactivity was also observed scattered in the stump mesenchyme. Jag1 immunoreactivity is completely absent in control sections.

Our analyses provided valuable, albeit limited, information about how the differential expression of miRNAs might be regulating gene expression during mid-bud blastema limb regeneration. However, profiling more time-points during limb regeneration is necessary in order to visualize the dynamics of the miRNA expression through the regeneration stages of wound closure, blastema formation and growth, early and late palette stages, as well as initiation of digit formation to completion of paw and limb regrowth. It is expected that as in other systems we will see stage-specific miRNA expression patterns.

We present a snapshot of the miRNA expression during a specific stage in the process of salamander limb regeneration. We demonstrated that *Amex-miR-21* is over-expressed in mid-bud blastemas and directly targets *Amex-Jag1* via the single *miR-21* target site present in its 3′-UTR, which is highly conserved. The functional significance of the pairing between *miR-21* and *Jag1* during limb regeneration has yet to be addressed. *In vivo* assays in which the expression and activity of either (or each) member of this couple is perturbed should provide answers. In the meantime, it is tempting to speculate that in the same way that *Hsa-Jag1* has been shown to be targeted by *Hsa-miR21* during monocyte-derived dendritic cell differentiation [48]; *Amex-Jag1* is being targeted by *Amex-miR-21* during mid-bud blastema limb regeneration. A unifying aspect of these two processes is the transition from a proliferative undifferentiated cell state to a differentiated stage when important cell fate commitments are being determined. We anticipate that inhibition of *Amex-miR-21* by exogenous antiMirs, or the addition of *JAG1*, could block blastemal cell differentiation in a functional manner. Thus, we hypothesize that *JAG1*, which in pathological contexts is found overexpressed in highly proliferative cells, is downregulated during limb regeneration by the highly expressed *miR-21* that targets its 3′-UTR recognition site. Therefore, proliferating blastemal cells previously under the influence of *JAG1* can become free to commit to their cell fate, as part of their transition to advanced regeneration stages (i.e., palette and digit stages).

## Materials and Methods

### Animals

All axolotls (*Ambystoma mexicanum*) were either bred at Yale University or obtained from the *Ambystoma* Genetic Stock Center at the University of Kentucky. Amputations and tissue collections were performed on animals measuring 10–15 cm from snout to tip of tail. Animals were anesthetized in 0.1% MS222 solution (Ethyl 3-aminobenzoate methanesulfonate salt, Sigma-Aldrich, St. Louis, MO, USA). Animal care and surgical procedures followed standard practices approved by the Yale University Institutional Animal Care and Use Committee (IACUC protocol number: 2011-10557).

### Cell culture

AL-1 cells were obtained from Stéphane Roy at the Université de Montréal and were cultured in Leibowitz L-15 medium which had been adjusted to 70% of its original osmolarity using autoclaved, filtered water. This amphibian osmolarity-adjusted basal medium was further supplemented with 10% fetal bovine serum, 10 µg/ml insulin and 1% penicillin-streptomycin (Invitrogen). Cells were maintained on gelatin-coated tissue culture plastic at a temperature of 25°C under normal atmospheric conditions.

### Microarray analysis of microRNAs

The hindlimbs of three white axolotls (medium to large animals) were amputated, and 0.5–1 cm long stump samples (including full thickness skin, bone, muscle, nerve fibers, etc) were collected under RNAse-free conditions and stored in RNAlater (Ambion, Foster City, CA) prior to RNA isolation. Seventeen days later (17 dpa, days-post-amputation), blastemas from the same animals were collected and stored in similar conditions than the stump tissues. Total RNA (including microRNAs) was collected from the samples using the mirVana miRNA Isolation Kit (Ambion). After running the RNA samples on a gel and confirming their integrity, 1.2 µg were sent to LC Sciences (Houston, TX) to be processed using their MicroRNA detection Microarray Service, and to be hybridized to a custom vertebrates chip (MRA-2001). This chip contained 3,918 selected vertebrate microRNAs (from human, opossum, Zebrafish, chicken, and frogs) based on the Sanger miRBase Release 12. Each probe was printed at least twice in the chip. An aliquot (5 µg) from the same total RNA isolation was sent to Exiqon Life Sciences (Woburn, MA) to be processed and hybridized to their All Species Array. This chip contained more than 2,000 captured probes complementary to microRNAs from vertebrates, invertebrates, plants and viruses reported in the mirBase v.9.2 database. It is important to note that each of these companies uses a different platform, and protocols to perform microarray analysis of microRNAs. Thus, while LC Sciences used *in situ* synthesized probes and μParaflo microfluidic chips to hybridize the RNA samples, Exiqon designed LNA probes that were later captured or attached to glass slides to be hybridized to the same axolotl RNA samples. Analysis of microarray datasets (QC assessments, background subtraction, Lowess normalization, and statistical tests) was also performed by LC Sciences and Exiqon according to their own standardized procedures. Upon receiving both datasets, differentially detected signal sets with *p*≤0.01 (LC Sciences), and *p*≤0.001 (Exiqon) were considered statistically significant to compensate for known differences in stringency criteria of detection call for each microarray platform [41].

### Expression analysis by Northern blot hybridization

For size determination of the axolotl *miR-21*, total RNA (including microRNAs) was collected from blastema and stump samples using the mirVana miRNA Isolation Kit (Ambion). Also, blood samples were collected from axolotls right after limb amputation using 10% ethylenediaminetetraacetic acid (EDTA) as anticoagulant. We followed the recommended alternate protocol to extract total RNA (including small RNAs) from nucleated red blood cells from aquatic animals using the RiboPure-Blood Kit (Ambion). All total RNA isolated from limb tissues and blood was treated with TurboDNAse (Ambion) following manufacture's recommendations, precipitated overnight at −80°C, washed and resuspended in 1× RNAsecure Resuspention solution (Ambion). For Northern Blots we followed published protocols [54], [55] with modifications as follows: ten micrograms of total RNA per sample were dissolved in Gel Loading Buffer II (Ambion), heated at 95°C for 5 min, loaded onto denaturing 15% TBE-Urea denaturing, SequaGel Sequencing gel (National Diagnostics, Atlanta, GA) along with a 10 bp DNA ladder (Invitrogen, Carlsbad, CA), and transferred to a Zeta Probe plus membrane (Bio-Rad, Hercules, CA). Membranes were equilibrated with 2× SSC and prehybridized at 42°C for 1 h in ULTRAhyb-Oligo buffer (Ambion). Prior to hybridization, miRCURY™ LNA detection probes were labeled using DIG Oligonucleotide Tailing Kit 2^nd^ Generation (Roche Applied Science, Indianapolis, IN). DIG-labeled LNA probes complementary to either *hsa-miR-21* or *hsa-U6* (loading control) were hybridized to the membranes overnight at 37°C in ULTRAhyb-Oligo buffer (Ambion). Following hybridization, the membranes were washed twice for 30 min in NorthernMax Low Stringency wash solution no. 1 (Ambion) at 42°C, rinsed for 5 min in 1× Wash Buffer from the DIG wash and Block Buffer Set (Roche), blocked for 1 h in 1× Blocking Solution (Roche), incubated for 1 h in antibody solution (Anti-DIG-AP 1∶10,000 in 1× Blocking solution, Roche), washed twice for 15 min in 1× Wash Buffer, equilibrated by rinsing twice for 5 min with 1× Detection Buffer (Roche). Then, following instructions from the DIG Luminescent Detection Kit (Roche), blots were incubated with the chemiluminescent substrate for alkaline phosphatase CSPD (Roche) and exposed to Amersham Hyperfilm ECL (GE Healthcare Life Sciences, Piscataway, NJ).

### Expression analysis by real-time RT-PCR

Total RNA (including microRNAs) was collected from a new set of 17 dpa blastema and stump samples (three biological replicates) using the mirVana miRNA Isolation Kit (Ambion). RNA quality and amount was assessed using the Bioanalyzer 2100 (Agilent Technologies, Santa Clara, CA) and the NanoDrop ND-1000 (NanoDrop Technologies, Wilmington, DE) respectively. MicroRNAs were converted into cDNA by reverse transcription using microRNA-specific primers and the miRCURY LNA First-strand cDNA Kit (Exiqon). Then, the cDNA was amplified by real-time PCR using the human miCURY™ LNA microRNA PCR system and miRCURY LNA SYBR Green Master Mix (Exiqon). The experiment was performed with *miR-21* and five putative microRNA endogenous controls chosen from the previous microarray microRNA profiling data. The stability of the endogenous controls was evaluated and the best two endogenous controls were selected using the SLqPCR R-package. For every endogenous control gene, the pair-wise variation with all other endogenous controls was determined as a gene stability measurement M. An M value below 1.5 is recommended and genes with expression stability above 1.5 were considered unstable across the samples and unsuitable for endogenous controls in this experiment [43]. Two of the endogenous control candidates (*miR-20a* and *miR-200b*) were considered acceptable and used for normalizing the quantified signal (Cp) of the microRNAs. We considered the Cp value for each sample as the median of at least three out of four technical replicates with a standard deviation (SD) less than 0.5. The PCR efficiency was estimated from a serial dilution of cDNA generated from pooled RNA of the samples for each assay. The Cp values were scaled to the average of all Cp values of the unknown samples (endogenous controls and *miR-21*) and corrected for assay-specific PCR efficiency. A normalization factor was calculated based on the geNorm algorithm for the endogenous controls. The relative expressions (fold changes) were calculated based on the efficiency corrected ΔΔCt method [56]. This method is based on the comparison of the distinct cycle differences between the test microRNA and the endogenous controls in a sample, and the average Cp values of all the unknown samples. All qPCR analyses were performed on three biological replicates, each of them being measured four times. Negative controls containing all reagents except template were included on each reaction plate.

### RACE cloning of the *Ambystoma mexicanum* 3′-UTR Jagged1 gene

The full length 3′-UTR (1,464 bp) of the *A. mexicanum Jagged1* (*Amex-Jag1*, accession number: JF907581) was cloned from axolotl stump cDNA using rapid amplification of cDNA ends (RACE) PCR [57]. RACE primers were designed by using partial preliminary axolotl sequence information that was obtained by aligning the human with the *Xenopus laevis* 3′-UTR of *Jagged1*, and finding BLAST matches with publicly available short 454-cDNA sequencing reads and expressed sequence tags (ESTs) from axolotl deposited in the Sal-Site at http://www.ambystoma.org [58], [59]. All sequence reactions were performed by the DNA Analysis Facility on Science Hill at Yale University.

### Construction of luciferase reported vectors

PCR amplified *Amex-Jag1* 3′-UTR of confirmed sequence, containing one *miR-21* target site, was cloned into the XhoI-NotI restriction sites of the psiCHECK-2 vector (Promega, Madison, WI). This vector has been widely used to examine the effect of 3′-UTRs, such as miRNA target sequences, on gene expression. It contained a multiple cloning region downstream of the stop codon of an SV40 promoter-driven *Renilla* luciferase gene. Due to the nominal activation of SV40 in amphibian cells (unpublished observation), we modified the psiCHECK-2 vector by substituting in a CMV promoter in its place. This allowed for strong expression of a *Renilla* transcript with the *Amex-Jag1* 3′-UTR sequence in transfected amphibian cells. *Renilla* luciferase activity was then used as a reporter to assess the effect of the 3′-UTR on transcript stability and translation efficiency in the presence/absence of *miR-21*. psiCHECK-2 also contains a constitutively expressed firefly luciferase gene to normalize transfections, thereby eliminating the need to transfect a separate co-reporter. In addition to psiCHECK-2 vector containing the *Amex-Jag1* 3′-UTR, we constructed a biosensor containing only the 22 nut target site for the human *miR-21*. This miR-21 biosensor was made by aligning sense and anti-sense oligonucleotides containing the *Hsa-miR-21* target sequence and 5′-XhoI and 3′-NotI overhangs for cloning into the respective sites in the psiCHECK-2 vector downstream of the *Renilla* luciferase gene (sense oligo: 5′- tcgagTCAACATCAGTCTGATAAGCTAgc -3′; antisense oligo: 5′- ggccgcTAGCTTATCAGACTGATGTTGAc). Moreover, seedless mutants (lacking the 7 nut complementary to the *miR-21* seed) of the psiCHECK-2*-Amex-Jag1_*3′-UTR vector and the psiCHECK-2*-Target-of-Hsa-miR-21* vector, were generated via site-directed mutagenesis using the QuikChange II site-directed mutagenesis kit (Agilent Technologies, Santa Clara, CA). The sequence of each of the vectors used for luciferase assays was carefully confirmed at least twice before performing the actual assays.

### Transfections and luciferase assays

AL-1 axolotl dermal fibroblasts were a gift from Stéphane Roy (Université de Montréal). The cells were electroporated with the luciferase vectors and Pre-miRs as noted in **Figure 4.B** using an Amaxa nucleofector device (Lonza - Basel, Switzerland). AL-1 cells (200,000 per test condition) were pulse-electroporated in a 100 µl reaction volume with 2 µg psiCHECK-2 plasmids containing different biosensors of miRNA activity and then plated out into 5 ml of culture medium; where noted, 2 nmol of Pre-miR (mimics of miRNAs) were also added to the electroporation reactions. Additionally, a small amount (∼20 ng) of an unrelated vector expressing a red fluorescent protein was co-transfected on each well to serve as visual assessment of transfection success (typically 60 to 80%). The cells were assayed for luciferase activity 48 h post transfection using the Dual-Glo Luciferase Assay System following manufacture's protocol (Promega, Madison, WI). Luminescence levels were measured using a Wallac Victor^2^ 1420 Multilabel Counter (Perkin Elmer, Waltham, MA). The *Renilla* reporter data was normalized to the Firefly co-reporter data and the ratios analyzed as percentage of activity in relation with their respective control (e.g., *Amex-miR-21* vs. *Amex-miR-21*+Pre-miR-21).

### Immunohistochemistry

Regenerating limbs at 17 dpa were collected and fixed in 4% paraformaldehyde containing 5% sucrose overnight at 4°C. Tissues were rinsed three times for 15 min each in 0.8× PBS and left for 2–3 days rocking at 4°C in decalcifying solution (18% EDTA in 0.8× PBS, 0.07% glycerol, 5% sucrose). Decalcified limbs were transferred to a 30% sucrose/0.8× PBS solution and left rocking overnight at 4°C. Next day, tissues were transferred to fresh sucrose solution and left at 4°C without rocking for 6 h or until the samples sunk to the bottom of the tubes. Samples were left overnight at 4°C without rocking in a 50∶50 degassed solution of 30% sucrose/0.8× PBS. Tissues were embedded in Optimal Cutting Temperature compound (OCT; Tissue Tek; Sakura Finetek, Torrance, CA), and cryostat sections (9 µm) were mounted in Superfrost Plus microscope slides (Erie Scientific, Portsmouth, NH). The indirect immunofluorescence method was followed [60]. Slides were air dried and blocked for 1 h in 1∶50 donkey normal serum (Santa Cruz Biotechnology, Santa Cruz, CA). After permeabilization in 0.5% Triton X-100 in PBS, and PBS washes, goat polyclonal primary antibody against human Jagged1 (sc-6011, Santa Cruz Biotechnology) was used at 1∶100 dilution and left in a humid chamber for 24 h at room temperature. Next day, slides were washed 3×15 min in PBS, and the secondary antibody, Alexa fluor 594 donkey anti-goat IgG (H+L) (Invitrogen) was administered in a 1∶50 dilution and left for 1 hr in a humid chamber. After 3×15 min washes in PBS, slides were mounted in VECTASHIELD HardSet mounting medium with DAPI (Vector Laboratories, Burlingame, CA). Negative controls included the use of normal serum as primary antibody in some slides, preincubation of the primary antibody with its corresponding blocking peptide (sc-6011P, Santa Cruz Biotechnology) for 2 h in order to neutralize the antibody before its addition to the sections. No Jagged1 immunoreactivity was detected in negative control slides. Dilution of serum and antibodies was done in RIA Buffer (0.05 M potassium phosphate buffer (pH 7.4) containing 0.5% BSA and 0.01% Na azide). Sections were examined in a Zeiss AxioImager M1 fluorescence microscope (Carl Zeiss MicroImaging, Thornwood, NY) equipped with a CCD camera (AxioCam MR3; Carl Zeiss). To obtain an image of the whole regenerating limb, photos of 21 overlapping fields of view were taken at 10× magnification, and the final image was manually reconstituted from its component overlapping images using Adobe Photoshop CS (Adobe Systems Inc, USA).

## Supporting Information

File S1 **MiRNA-microarray results obtained using the LC Sciences and Exiqon platforms.** Multiarray normalized data, fold changes and statistical tests are included. Probes targeting *miR-21* from different species being upregulated in salamander limb regenerating blastemas are highlighted.
(XLS)
